# Effect of pharyngeal musculature and genioglossus exercising on obstructive sleep apnea-hypopnea syndrome following uvulopalatopharyngoplasty

**DOI:** 10.3389/fpsyt.2026.1786737

**Published:** 2026-05-05

**Authors:** Pingping Zhang, Tongfei Qu, Xiaona Zhang, Runze Zhu

**Affiliations:** Department of Otorhinolaryngology, The First People’s Hospital of Linping District, Hangzhou, Zhejiang, China

**Keywords:** exercise, genioglossus muscle, obstructive sleep apnea-hypopnea syndrome, pharyngeal cavity, uvulopalatopharyngoplasty

## Abstract

**Objective:**

This study aimed to evaluate the effectiveness of pharyngeal musculature and genioglossus exercising as a postoperative rehabilitation intervention for patients with obstructive sleep apnea-hypopnea syndrome (OSAHS) following uvulopalatopharyngoplasty (UPPP)—a setting with limited prior evidence.

**Methods:**

This is a retrospective cohort study conducted in the first people’s Hospital of Linping District, Hangzhou. They included 120 patients of OSAHS who received UPPP between October 2022 and October 2024. Sixty patients who received pharyngeal and genioglossal muscle exercises were matched with the cohort who did not receive any exercise in a 1:1 ratio. The main outcome was the clinical efficacy 6 months after operation. The secondary outcomes were the changes of apnea hypopnea index (AHI), lowest oxygen saturation during sleep (LSaO2), Pittsburgh sleep quality index (PSQI), and the World Health Organisation Quality of Life tool (WHOQOL-BREF) score.

**Results:**

Six months after operation, the clinical effective rate of the exercise group was significantly higher than that of the non-exercise group (*p* < 0.05). Before operation, there was no significant difference in AHI, LSaO2, PSQI and WHOQOL-BREF scores between the two groups (all *p* > 0.05). Six months after operation, the AHI,LSaO2, PSQI and WHOQOL-BREF scores of the two groups were significantly improved, and the AHI, LSaO2, PSQI, physical and psychological scores of the exercise group were better than those of the non-exercise group (all *p* < 0.05); However, there was no significant difference in the scores of environment and social domains between the two groups (all *p* > 0.05).

**Conclusions:**

Pharyngeal musculature and genioglossus exercising may improve postoperative outcomes and quality of life in patients undergoing UPPP, and could be considered a promising rehabilitation strategy in clinical practice.

## Introduction

​Obstructive sleep apnea-hypopnea syndrome (OSAHS) is a recurrent upper airway collapse and obstruction in sleep disorders (1, 2). OSAHS can cause hypoxemia and sleep structural disorder at night, which can lead to multiple system complications. Such as cardiovascular and cerebrovascular diseases, metabolic disorders and cognitive dysfunction, which seriously reduce the quality of life of patients (2, 3). It was reported that the prevalence of OSAHS among adults in China was 11%, and was found to increase with rising body mass index (BMI) (3). OSAHS has become a serious public health problem (4).

However, several studies have shown that although UPPP has short-term efficacy, its long-term effectiveness is often compromised (5, 6). Recurrence of OSAHS postoperatively may be attributed to factors such as upper airway tissue relaxation, postoperative scarring, weight gain, or progression of neuromuscular dysfunction over time (7). A recent 8-year follow-up study by Sundman et al. (8) demonstrated a significant rebound in apnea–hypopnea index (AHI) several years after UPPP, especially in patients with high BMI or increasing weight post-surgery.

Meanwhile, oropharyngeal muscle training has gained increasing attention as a non-invasive intervention in OSAHS populations. In a recent randomized controlled trial, Marzouqah et al. (9) applied structured oropharyngeal exercises to post-stroke OSAHS patients and reported significant improvements in AHI, oxygen desaturation, and sleep quality. Although these findings support the efficacy of targeted upper airway exercises, evidence for their use in the postoperative recovery phase of UPPP remains limited. Our study addresses this gap by investigating the clinical effects of pharyngeal and genioglossus muscle training as a postoperative rehabilitation strategy in patients undergoing UPPP.

Uvulopalatopharyngoplasty (UPPP) is one of the most commonly used surgical procedures for the treatment of OSAHS. He et al. (6) conducted a meta-analysis of UPPP related studies and found that UPPP can achieve good results both in the short-term and long-term after surgery. UPPP can improve the upper airway obstruction by removing part of the velopharyngeal tissue and enlarging the pharyngeal cavity (6, 10). However, the relaxation and collapse of pharyngeal muscles (such as palatoglossus and genioglossus) will occur again due to scar contraction, poor recovery of muscle function and weight gain, especially in obese patients (6, 10, 11). Therefore, how to improve the efficacy of UPPP and reduce the recurrence rate of adjuvant therapy has become an urgent problem to be solved. Brown et al. (12) also found that the decrease of upper airway muscle tension and strength was the main cause of OSAHS; In addition, Braga et al. (13) showed that the greater the anterior lingual muscle strength measured before UPPP surgery, the better the surgical effect. This indicates that increasing the physiological tension and strength of the pharyngeal muscles and genioglossal muscles may help to maintain the patency of the pharyngeal cavity after UPPP, thereby improving the efficiency of surgical treatment (12–14).

At present, the clinical evidence-based evidence about the influence of pharyngeal cavity and genioglossal muscle functional exercise on the effect of UPPP is still weak. Therefore, the clinical data of pharyngeal cavity and genioglossal muscle function exercise in patients with OSAHS after UPPP operation were retrospectively analyzed. In order to provide new ideas for the selection of surgical methods and personalized and precise treatment of OSAHS patients.

## Materials and methods

### Patients

**Figure 1:**
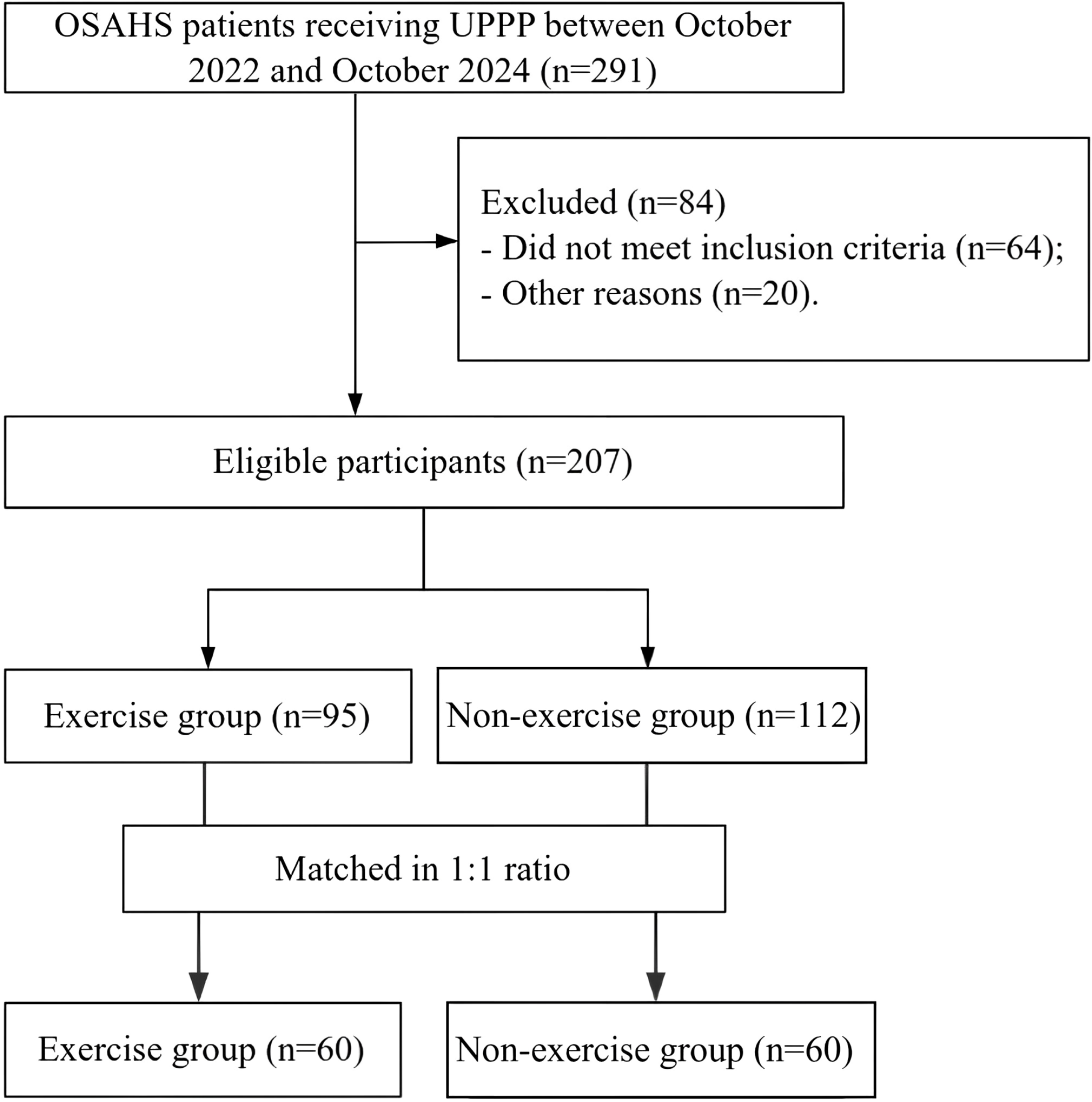
Patient screening flow chart. UPPP, uvulopalatopharyngoplasty; OSAHS, obstructive sleep apnea-hypopnea syndrome.

This is a retrospective cohort study conducted in the first people’s Hospital of Linping District, Hangzhou. The study enrolled 291 patients with OSAHS and 84 cases were excluded after screening. A total of 120 patients with OSAHS who received UPPP between October 2022 and October 2024 were ultimately included (**Figure 1**). Sixty patients who received pharyngeal and genioglossal muscle exercises were matched with the cohort who did not receive any exercise in a 1:1 ratio based on gender, age and BMI. Education level, neck circumference, apnea hypopnea index (AHI) and lowest oxygen saturation during sleep (LSaO2). AHI and LSaO2 were recorded by polysomnography (PSG) (Tyco Sandman polysomnography respiratory monitoring system). All patients underwent two overnight PSG assessments: the first at baseline (prior to UPPP), and the second at 6 months postoperatively. The exercise training began approximately 1 month after UPPP, when the surgical site was confirmed to be healed. The 6-month PSG was scheduled after a 5-month training period, with both tests conducted in the same sleep laboratory using standardized procedures.

### Inclusion criteria

- It meets the diagnostic criteria of OSAHS (1);
- AHI > 15 times/hour, LSaO2<85% (15);
- 18–65 years old;
- The occlusive plane was in oropharyngeal region;
- The clinical data are complete.

### Exclusion criteria

- Patients with central sleep apnea syndrome;
- Severe cardiovascular and cerebrovascular diseases;
- Airway obstruction was not at oropharyngeal level;
- Scar constitution;
- Overlap syndrome;

According to the 2011 revised Guidelines for the Diagnosis and Treatment of Obstructive Sleep Apnea–Hypopnea Syndrome (OSAHS) issued by the Sleep-Disordered Breathing Group of the Chinese Thoracic Society (Chinese Medical Association), moderate OSAHS is defined as an AHI >15 to ≤30 events per hour; an AHI of exactly 15 events per hour falls within the mild range (15). Therefore, the inclusion criterion of AHI ≥ 15 was set to ensure that only moderate to severe OSAHS patients were enrolled, which is consistent with the indications for UPPP. In addition, all patients had a lowest oxygen saturation (LSaO2) < 85%, further confirming disease severity. In fact, the median AHI of both groups exceeded 40, indicating that the vast majority of included patients were in the severe category. This inclusion threshold is widely accepted in clinical research and has been adopted in previous studies and surgical outcome evaluations for OSAHS (5).

### Preoperative airway assessment

All patients underwent a standardized preoperative evaluation before UPPP. This included a detailed clinical history, physical otolaryngologic examination, and overnight polysomnography to confirm the diagnosis and severity of OSAHS. Upper airway obstruction at the oropharyngeal level was assessed by experienced otolaryngologists using awake nasopharyngoscopy in all patients, and Müller’s maneuver was performed in selected cases to further evaluate dynamic pharyngeal collapse. Drug-induced sleep endoscopy (DISE) was not routinely performed due to resource limitations, but surgical candidacy for UPPP was determined based on the comprehensive clinical and endoscopic findings. In this study, we used UPPP rather than other pharyngoplasty techniques such as barbed reposition pharyngoplasty (BRP) and expansion sphincter pharyngoplasty (ESP), due to resource limitations and economic considerations.

### Data collection

The following clinical data were collected for all participants at baseline and 6 months postoperatively: (1) demographic characteristics including age, gender, body mass index (BMI), neck circumference, and education level; (2) polysomnography parameters such as apnea–hypopnea index (AHI) and lowest oxygen saturation during sleep (LSaO2); (3) sleep quality scores measured by the Pittsburgh Sleep Quality Index (PSQI); (4) health-related quality of life scores assessed by the WHOQOL-BREF questionnaire; (5) treatment response categorized by AHI-based efficacy grading.

### UPPP

General anesthesia via nasal intubation. The fat, bilateral tonsils, palatoglossal arch and palatopharyngeal arch in the palatine velum space were excised. The palatopharyngeal muscle was suspended and sutured; Tension free contraposition suture of mucosa; Truncate the hypertrophic uvula; Enlarge the left-right and anterior posterior diameters of pharyngeal cavity. After the operation, the patients were instructed to reduce weight, quit smoking and alcohol, and try to sleep on their sides only. All surgeries were performed by the same otolaryngology team using a standardized surgical technique, ensuring procedural consistency across patients.

### Pharyngeal musculature and genioglossus exercising

The terminology “*pharyngeal musculature and genioglossus exercising*” was selected to reflect the anatomical and functional regions targeted by the intervention, including the genioglossus, palatoglossus, pharyngeal constrictors, and buccinator muscles. These exercises were designed to enhance neuromuscular tone in the upper airway and simulate functional actions such as breathing and swallowing.

One month after UPPP, if the suture at the surgical site fell off cleanly, the mucosa recovered well, and there was no bleeding, pain, or swallowing discomfort, the exercise program was initiated. Patients were instructed to open their mouth and extend their tongue 10 times each morning and evening. Submental massage was performed using the thumb pulp for 1 minute, followed by forward and upward chin pressure with the thumb for 10 repetitions (5 seconds each). Patients were also asked to perform 10 cheek-puffing maneuvers (buccal ballooning), where the cheeks were puffed while the lips were closed, holding each for 5 seconds. Then, they pinched their nose and closed their mouth 10 times (5 seconds each), followed by deep nasal breathing with the mouth closed for 20 repetitions. These exercises were selected based on previous studies that demonstrated their effectiveness in targeting upper airway dilator muscles (16, 17). Specifically, they activate the genioglossus, palatoglossus, platysma, and pharyngeal constrictors. These maneuvers simulate tongue protrusion, soft palate movement, and pharyngeal wall tightening, which are crucial for maintaining airway patency. Regular repetition aims to strengthen muscle tone, reduce airway collapsibility, and improve sleep-related respiratory function. After each training session, patients were required to submit photos or videos via the WeChat application as daily check-ins. Medical staff reviewed these submissions weekly to evaluate compliance, provide feedback, and issue reminders as needed. This approach ensured objective documentation and continuous supervision. Patients were also followed up at least once a month to monitor progress and reinforce adherence.

### Main outcome ​

The main outcome was clinical efficacy. 1) Cured (AHI < 5 times/h); 2) Markedly effective (AHI < 20 times/h and the decreased range ≥ 50%); 3) Effective (AHI decreased ≥ 50%, but not reduced to less than 20 times/h); 4) Ineffective (AHI decreased by<50%). Total effective rate= (cured + markedly effective + effective)/total number.

These efficacy thresholds were adapted from the “Guidelines for the Diagnosis and Treatment of Obstructive Sleep Apnea–Hypopnea Syndrome (OSAHS)” issued by the Sleep-Disordered Breathing Group of the Chinese Thoracic Society (Chinese Medical Association) and are also consistent with efficacy grading systems used in previous domestic studies (5). An AHI < 5/h is commonly accepted as a normalized level, while a ≥50% reduction is considered a clinically meaningful response in both Chinese and international literature (17). These criteria were selected to facilitate interpretation of therapeutic outcomes.

### Secondary outcomes

The secondary outcomes were AHI, changes in sleep quality and quality of life. 1) Pittsburgh sleep quality index (PSQI) was used to evaluate sleep quality: it was composed of 19 self-rated items and 5 other rated items, of which 18 items were composed of 7 factors, and each factor was scored according to the 0–3 score level; The cumulative score of each factor component is the total score of PSQI, and the total score range is 0–21 points; The higher the score, the worse the sleep quality. 2) The quality of life was assessed using the World Health Organisation Quality of Life tool (WHOQOL-BREF) score; The scale is divided into four dimensions: physical, psychological, environmental and social domains, with a single dimension of 0–100 points; The higher the score, the higher the quality of life.

### Statistical methods

We used SPSS statistical software Windows version 25.0 (IBM, USA) for statistical analysis. The baseline demographic and clinical characteristics of the two groups were compared by Pearson chi square test. According to the normality of the data, the independent sample t test and Mann Whitney test were used to compare the continuous variables. The normality of continuous variables is tested by Shapiro Wilks test. If the normality is violated, we use Mann Whitney test. For the measurement of classification results, we use Pearson chi square test (or Fisher exact test, if the expected frequency of any cell in the contingency table is less than 1) to compare the differences between groups. Double sided *p*  < 0.05 was considered statistically significant. In addition to hypothesis testing, effect sizes with 95% confidence intervals were calculated to quantify the magnitude of between-group differences. Between-group comparisons for continuous variables were performed using independent sample t-tests or Mann-Whitney U tests based on the normality of distribution. Effect sizes for normally distributed data are expressed as mean differences with 95% confidence intervals (CIs). For non-normally distributed outcomes, the Hodges–Lehmann median difference (HL MD) and its 95% CI are reported. For binary outcomes, we report risk ratios (RRs) with Katz 95% CIs. For r × c categorical variables, we report Cramér’s V with bootstrap 95% CIs. All effect sizes and their confidence intervals are presented in **Tables 1**–**4**. As this was a retrospective cohort study, no *a priori* power calculation was performed. Instead, all eligible patients during the study period were included, and 1:1 group matching was conducted based on key variables (gender, age, BMI, education level, neck circumference, AHI, and LSaO2) to improve comparability. In future prospective trials, formal sample size estimation will be conducted to enhance statistical rigor.

**Table 1:** Comparison of basic population characteristics between the two groups.

Index		Exercise group (n=60)	Non-exercise group (n=60)	HL MD/RR/V (95%CI)	*χ^2^/t/Z*	P
Gender, n (%)	Male	34 (56.7)	38 (63.3)	1.15 (0.80-1.64)	0.556	0.456
	Female	26 (43.3)	22 (36.7)			
Age (years), M(IQR)		38.5 (28.8, 52.0)	44.5 (35.0, 53.2)	-3.5 (-8.0-1.0)	-1.540	0.123
BMI (kg/m²), M(IQR)		26.35 (24.65-28.9)	26.3 (24.95-29.95)	-0.4 (-2.1-0.8)	-0.596	0.551
Education level, n (%)				0.050 (0.019-0.263)	0.300	0.861
Primary school		10 (16.7)	9 (15.0)			
Junior high school		29 (48.3)	32 (53.3)			
High school and above		21 (35.0)	19 (31.7)			
Neck circumference (cm), M(IQR)		41.0 (38.8, 44.0)	41.0 (37.8, 43.2)	1.0 (-1.0-2.0)	0.682	0.494
Baseline AHI (times), M(IQR)		42 (35-56.5)	41.5 (32.5-46.5)	3.0 (-1.0-8.0)	-1.211	0.226
Baseline LSaO2 (%), M(IQR)		71 (62.95-77.65)	74.85 (65.25-78.65)	-1.9 (-5.2-1.3)	-1.205	0.228

Continuous variables are presented as mean ± SD (if normally distributed; Student/Welch t test) or median (IQR) (Mann–Whitney U, Z reported). Effect sizes are reported as a single measure with 95% CI: Mean diff for t tests; HL MD (Hodges–Lehmann median difference) for Mann–Whitney; RR for 2×2 categorical; V (Cramér’s V) for r×c categorical. Differences are computed as exercise − non-exercise. Normality by Shapiro–Wilk; homogeneity by Levene. Chi-square uses the expected-count rule (≥80% cells ≥5 and all ≥1); otherwise Fisher exact test is used for 2×2 tables. HL MD CI via bootstrap (B = 1000); RR CI via Katz; V CI via bootstrap. AHI, apnea hypopnea index; LSaO2, lowest oxygen saturation during sleep.

**Table 2:** Comparison of clinical efficiency between the two groups​.

Group	Cured	Markedly effective	Effective	Ineffective	Total effective rate
Exercise group (n=60)	8 (13.3)	30 (50.0)	15 (25.0)	7 (11.7)	53 (88.3)
Non-exercise group (n=60)	4 (6.7)	24 (40.0)	14 (23.3)	18 (30.0)	42 (70.0)
RR(95% CI)					1.26 (1.04–1.53)
*χ^2^*					6.114
*P*					0.013

Values are n (%). Total effective rate = cured + markedly effective + effective. For the 2×2 comparison, Pearson’s chi-square is used when the expected-count rule is met (≥80% of cells ≥5 and all ≥1); otherwise Fisher’s exact test is applied. Effect size is the risk ratio (RR) with Katz 95% CI.

**Table 3:** Comparison of AHI, LSaO2 and PSQI scores between the two groups.

Index	Exercise group (n=60)	Non-exercise group (n=60)	HL MD/Mean diff (95%CI)	*Z/t*	P
Baseline					
AHI (times),M(IQR)	42 (35-56.5)	41.5 (32.5-46.5)	3.0 (-1.0-8.0)	-1.211	0.226
LSaO2(%),M(IQR)	71 (62.95-77.65)	74.85 (65.25-78.65)	-1.9 (-5.2-1.3)	-1.205	0.228
PSQI (score),M(IQR)	15 (12-17.5)	15 (13-18)	0.0 (-2.0-1.0)	-0.701	0.483
Postoperative					
AHI,M(IQR)	13 (8-24)^*^	20 (13-30)^*^	-5.0 (-9.0- -1.0)	-2.505	0.012
LSaO2(%),M(IQR)	84.6 (79.65-89.25)^*^	82.4 (72.5-86.7)^*^	2.6 (-0.2-5.8)	-2.197	0.028
PSQI, mean ± SD	10.6 ± 3.4^*^	12.4 ± 2.9^*^	-1.8 (-2.9- -0.6)	-3.054	0.003

Compared with before treatment in the same group, ^*^P<0.05. Between-group tests at each time point use Welch t for approximately normal data (otherwise Mann–Whitney U; Z reported). Effect size is reported as a single measure with 95% CI: Mean diff for t tests; HL MD (Hodges–Lehmann median difference) for Mann–Whitney U. AHI, apnea–hypopnea index; LSaO2, lowest arterial oxygen saturation; PSQI, Pittsburgh Sleep Quality Index; SD, standard deviation; M(IQR), median (IQR). Differences are computed as exercise − non-exercise.

**Table 4:** Comparison of WHOQOL-BREF scores between the two groups, M(IQR).

Index	Exercise group (n=60)	Non-exercise group (n=60)	HL MD (95%CI)	*Z*	*P*
Baseline					
Physical	53.6 (50-57.1)	53.6 (50-60.7)	0.0 (-12.3–10.7)	-1.175	0.240
Psychological	41.7 (37.5-50)	45.8 (37.5-52.1)	-4.1 (-20.8–16.7)	-1.213	0.225
Environmental	51.55 (50-56.3)	53.1 (50-56.3)	-1.6 (-12.5–9.4)	-0.765	0.444
Social	58.3 (41.7-58.3)	50 (41.7-58.3)	8.3 (-16.7–16.7)	-0.985	0.325
Postoperative					
Physical	78.6 (71.4-82.1)^*^	71.4 (67.9-78.6)^*^	7.2 (-7.2–17.9)	-3.456	0.001
Psychological	79.2 (70.8-79.2)^*^	70.8 (62.5-79.2)^*^	8.4 (-12.5–20.9)	-3.011	0.003
Environmental	71.9 (65.6-79.7)^*^	68.8 (62.5-78.1)^*^	3.1 (-12.5–18.8)	-1.656	0.098
Social	75 (66.7-83.3)^*^	66.7 (62.5-75)^*^	8.3 (-16.7–25.0)	-1.265	0.206

Values are expressed as median (interquartile range). Between-group comparisons at each time point were performed using the Mann–Whitney U test. Effect sizes are reported as the Hodges–Lehmann median difference (HL MD) with 95% confidence intervals. *p < 0.05 compared with baseline within the same group. WHOQOL-BREF domains: Physical, Psychological, Environmental, and Social. Differences are calculated as exercise cohort minus non-exercise cohort.

## Results

From October 2022 to October 2024, we enrolled 291 OSAHS patients who received UPPP, excluded 84 cases, and finally 207 cases met the conditions of this study. Among them, 95 cases received pharyngeal cavity and genioglossal muscle functional exercise after UPPP (exercise group), and 112 cases did not receive any exercise (non-exercise group). The two groups were matched in a ratio of 1:1, with 60 patients in each group. There was no significant difference in demographic characteristics and baseline AHI and LSaO2 scores between the two groups (all *p* > 0.05) (**Table 1**). Additionally, effect sizes for baseline comparisons are provided in **Table 1** to facilitate understanding of between-group equivalence prior to intervention.

As shown in **Table 2**, six months after operation, the clinical effective rate of the exercise group was 88.3% (53/60), and that of the non-exercise group was 70.0% (42/60). The clinical effective rate of the exercise group was significantly higher than that of the non-exercise group (*χ^2^* = 6.114, *p* < 0.05).​ The between-group risk ratio (RR) for clinical effectiveness was also calculated and is shown in **Table 2**, indicating a favorable effect of the intervention.

As shown in **Table 3**, there was no significant difference in baseline AHI, LSaO2, and PSQI levels between the two groups (all p > 0.05). At 6 months postoperatively, the exercise group showed significantly lower AHI and PSQI scores and higher LSaO2 compared to the non-exercise group (all p < 0.05). Importantly, the between-group effect size for AHI was a Hodges–Lehmann median difference of -5.0 events/h (95% CI: -9.0 to -1.0), and for PSQI was -1.8 points (95% CI: -2.9 to -0.6). These improvements are considered both statistically and clinically meaningful. LSaO2 also improved, with a median difference of 2.6% (95% CI: -0.2 to 5.8), approaching clinically relevant thresholds.

As shown in **Table 4**, there were no significant differences in baseline WHOQOL-BREF domain scores between the groups. At 6 months, both groups showed improvements across all domains (all p < 0.05). Inter-group analysis at 6 months revealed superior scores in the exercise group for the physical and psychological domains. The HL MD was 7.2 (95% CI: -7.2 to 17.9) for physical health and 8.4 (95% CI: -12.5 to 20.9) for psychological health. These results indicate a more pronounced functional and emotional recovery in the intervention cohort compared to the control group. These differences reflect meaningful functional and emotional benefits. No significant between-group differences were found in environmental and social domain scores.

## Discussion​

Our findings demonstrated that patients who engaged in pharyngeal musculature and genioglossus exercising after UPPP achieved significantly greater clinical efficacy, improved sleep quality, and better quality of life compared to those who did not exercise. These improvements were reflected across AHI, PSQI, and WHOQOL-BREF scores, aligning with previous studies reporting benefits of upper airway muscle training in OSAHS management (16, 18, 19).

From a pathophysiological perspective, the beneficial effects of pharyngeal musculature and genioglossus exercising observed in our study are likely mediated by improvements in upper airway neuromuscular function. Repetitive activation of the genioglossus, soft palate, and pharyngeal constrictor muscles may increase baseline muscle tone and endurance, enhance coordination between dilator muscles, and thereby reduce pharyngeal collapsibility during sleep. These mechanisms are consistent with previous randomized and feasibility trials showing that oropharyngeal muscle training can reduce AHI, improve oxygenation, and enhance sleep-related quality of life in patients with moderate OSAHS or post-stroke OSAHS (9, 17). Strengthening the upper airway dilator muscles may thus represent a physiologically targeted and non-invasive strategy to support postoperative airway stability after UPPP.

In addition to statistical significance, the observed differences demonstrated meaningful effect sizes with clinical implications. For example, the exercise group showed a median AHI reduction of 5.0 events/h compared to the non-exercise group (HL MD: -5.0; 95% CI: -9.0 to -1.0), which may correspond to a shift in OSAHS severity classification for many patients. The PSQI score also improved significantly (mean difference: -1.8; 95% CI: -2.9 to -0.6), which exceeds the threshold typically considered meaningful for subjective sleep quality improvement. Furthermore, improvements in WHOQOL-BREF domains, especially in the physical (HL MD: 7.2; 95% CI: -7.2 to 17.9) and psychological (HL MD: 8.4; 95% CI: -12.5 to 20.9) dimensions, suggest potential gains in patients’ functional recovery and psychological well-being, although the corresponding effect-size estimates should be interpreted cautiously. These results support the practical value of pharyngeal musculature and genioglossus exercising as a postoperative adjunct therapy after UPPP.

The oropharyngeal exercise protocol adopted in our study was based on prior evidence from Tang et al. (16), Guimarães et al. (17), and others, which targeted upper airway dilator muscles such as the genioglossus, palatine, and platysma. These exercises were initiated one month after UPPP when wound healing was confirmed. The intervention was simple, noninvasive, and supported by daily WeChat-based adherence monitoring. The potential mechanism may involve enhanced muscle tone and reduced pharyngeal compliance, contributing to postoperative airway stability (16, 17, 19–22). We recommend implementing such exercises for at least three months after UPPP to support functional recovery and long-term efficacy maintenance (23–25).

However, there was no significant difference in the scores of environmental and social domains between the two groups at 6 months after operation. This may be related to the selection of subjects, exercise methods and intensity, follow-up time and other factors. Six months after surgery, it may only cover the short-term rehabilitation stage, while the overall improvement of social function (such as returning to work, social circle reconstruction) usually lags behind the recovery of physiological function (26, 27). The environmental field may be dominated by personal and family economic conditions. In addition, Friberg et al. (28) showed that one third of patients had side effects after UPPP, including mucus, voice and dysphagia, especially elderly patients. However, this study did not analyze the side effects and recurrence rate of patients. This is mainly because the beginning of pharyngeal musculature and genioglossus exercising was reviewed one month after operation, excluding patients with bleeding, pain, dysphagia and so on.

Although this study confirmed that pharyngeal musculature and genioglossus exercising effectively improved the therapeutic outcomes of UPPP in patients with OSAHS, several limitations must be acknowledged. First, the current study was a single-center, retrospective analysis with a relatively small sample size, which may limit the generalizability of the findings. Second, although improvements in AHI, LSaO2, and quality of life were observed, no direct assessments of muscle-level function (e.g., tongue pressure or electromyography) were performed. This restricts our ability to elucidate the physiological mechanisms underlying the clinical benefits. Third, the exercise intervention was patient-led, and individual differences in execution or compliance may have affected outcome consistency. Fourth, while the intervention appeared effective in the short term, long-term outcomes, including recurrence rates after UPPP, remain unclear. Muscle adaptation may require extended time, and further studies with prolonged follow-up are needed to assess sustained efficacy. Fifth, we acknowledge that clinical efficacy definitions based on AHI reduction are not yet standardized internationally, which may limit cross-study comparisons. Finally, DISE wasn’t performed due to resource limitations. In the future, well-designed, multi-center, prospective randomized controlled trials are warranted to validate these findings and to explore the use of oropharyngeal muscle training as a standardized postoperative rehabilitation strategy for OSAHS patients following UPPP.

## Conclusion

Pharyngeal musculature and genioglossus exercising following UPPP may improve AHI, sleep quality, and overall quality of life in patients with OSAHS. While these findings are promising, the retrospective and single-center design limits generalizability. Future large-scale, high-quality prospective studies are needed to validate these results and assess long-term outcomes.

## Data Availability

The raw data supporting the conclusions of this article will be made available by the authors, without undue reservation.

## Glossary

OSAHS: obstructive sleep apnea-hypopnea syndrome
UPPP: uvulopalatopharyngoplasty
AHI: apnea hypopnea index
LSaO2: lowest oxygen saturation during sleep
PSQI: Pittsburgh sleep quality index
WHOQOL-BREF: World Health Organisation Quality of Life tool
BMI: body mass index.
